# Gene expression patterns in multiple organs in experimentally induced *Staphylococcus aureus *sepsis in pigs

**DOI:** 10.1186/cc12994

**Published:** 2013-11-05

**Authors:** Helle G Olsen, Morten A Rasmussen, Kerstin Skovgaard, Páll S Leifsson, Henrik E Jensen, Peter MH Heegaard, Ib M Skovgaard, Mads Kjelgaard-Hansen, Ole L Nielsen

**Affiliations:** 1 Department of Veterinary Disease Biology, University of Copenhagen, Frederiksberg, Denmark; 2 Department of Food Science, University of Copenhagen, Frederiksberg, Denmark; 3 Innate Immunology Group, National Veterinary Institute, Technical University of Denmark, Frederiksberg, Denmark; 4 Department of Mathematical Sciences, University of Copenhagen, Denmark; 5 Department of Small Animal Clinical Sciences, University of Copenhagen, Frederiksberg, Denmark

## Background

Animal research in sepsis needs analytical tools that can capture and exploit the complexity of the condition. To summarise the disease progression in a porcine model of severe *Staphylococcus aureus *sepsis, we used principal component analysis (PCA) as a multivariate approach to identify early dynamic expression patterns of 34 selected genes in the liver, lung, and spleen tissue.

## Materials and methods

We combined data from two related experimental studies in pigs haematogenously infected with a porcine pathogenic strain of *S. aureus *[1,2]. Seventeen infected pigs were euthanised at the following time points post infection (p.i.): 6 hours (*n *= 3), 12 hours (*n *= 3), 24 hours (*n *= 3), 30 hours (*n *= 1), 36 hours (*n *= 2), and 48 hours (*n *= 5). Five healthy controls were managed in parallel. Gene expression of 34 genes related to acute inflammation and haemostasis was measured in the liver, lung, and spleen by quantitative real-time PCR. The data matrix of 22 samples and 102 (34 × 3) variables were log-transformed, scaled to unit variance, and subjected to PCA.

## Results

**Figure 1:**
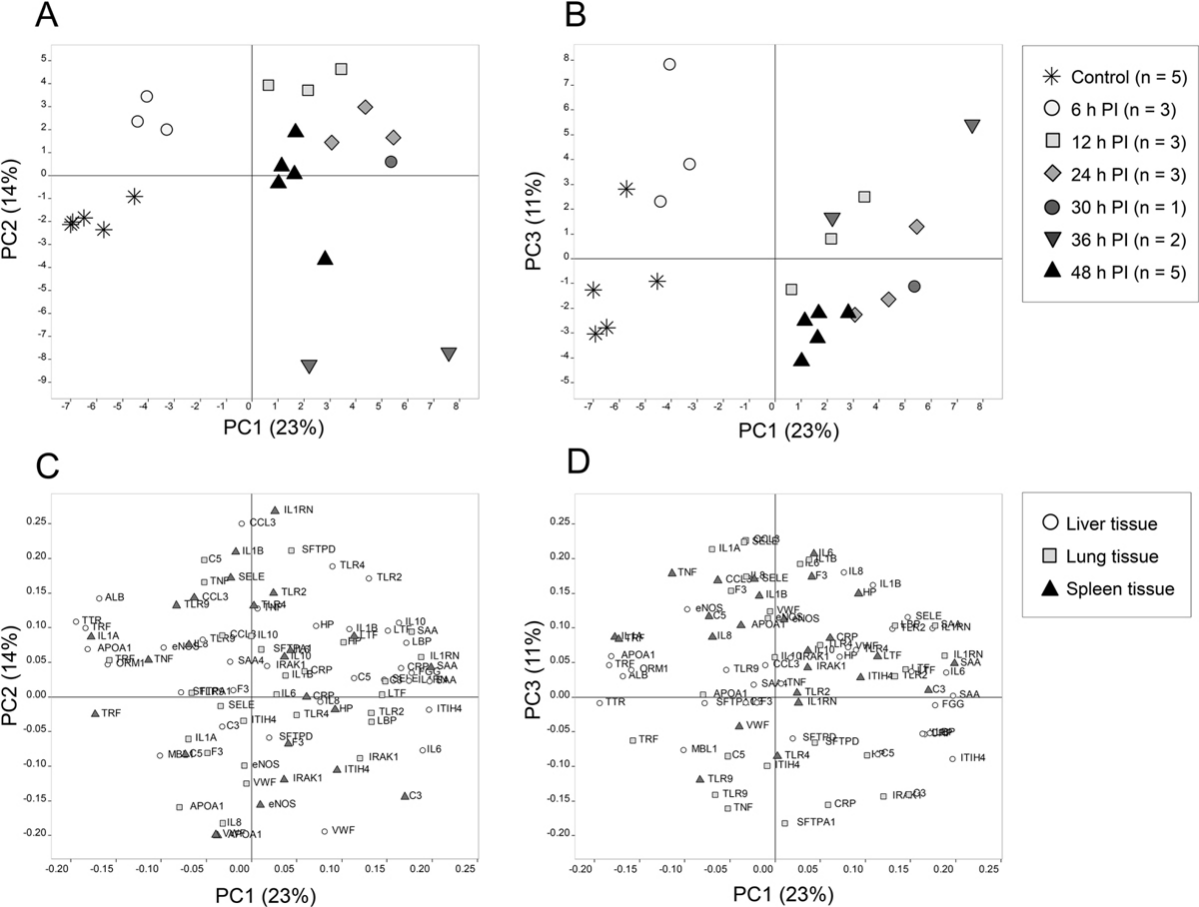
**Pigs (scores) and genes (loadings) in PC1-PC2 (A, C) and PC1-PC3 space (B, D)**. (A) Increased PC1 scores are seen in infected pigs from 12 hours until the end of the study (48 hours), with a peak around 24 hours. PC2 scores are increased after 6 hours of infection, with a peak at 12 hours. Thereafter, scores decline towards basal levels, or below. (B) PC3 scores are increased mainly in infected pigs at 6 hours p.i. (C) Genes with largest influence on the PC1 axis are mainly hepatic positive and negative acute phase genes (for example, SAA, ITIH4, TTR, TRF). Genes with largest influence on the PC2 axis are not specifically related to a single tissue. (D) The main influencers of the PC3 axis are inflammatory genes measured in lung tissue. Gene symbols are used according to the National Center for Biotechnology Information (NCBI).

Three (PC1 to PC3) distinct dynamic response patterns were identified. PC1: hepatic positive and negative acute-phase genes were the main influencers of a protracted pattern induced between 12 and 48 hours of infection, which explained 23% of the total variation in the dataset (Figure 1A, C). PC2: an acute pattern distinguished infected pigs from controls already after 6 hours and peaked around 12 hours p.i. After 30 to 48 hours, pigs had either reverted back to basal levels (*n *= 7) or below basal levels (*n *= 2) (Figure 1A). This pattern explained 14% of the total variation and was influenced by a systemic (nonorgan-specific) mixture of proinflammatory, anti-inflammatory and haemostatic genes (Figure 1C). The two pigs with low PC2 levels had suffered from overt disseminated intravascular coagulation when euthanised [3], and this outcome was clearly reflected by PC2. PC3: a per-acute pattern, influenced mainly by pulmonary proinflammatory genes (explaining 11% of the total variation), was induced in infected pigs at 6 hours p.i., while at later time points most pigs had moved towards basal levels (Figure 1B, D).

## Conclusions

Multivariate analysis (PCA) identified three temporally distinct patterns in gene expression data from the liver, lung, and spleen tissue: pulmonary inflammation was rapidly induced, followed by transient induction of a generalised inflammatory and haemostatic response, and initiation of the hepatic acute-phase response.
